# Tumor-infiltrating macrophages express interleukin-25 and predict a favorable prognosis in patients with gastric cancer after radical resection

**DOI:** 10.18632/oncotarget.7095

**Published:** 2016-01-31

**Authors:** Jinqing Li, Yuan Liao, Tong Ding, Bo Wang, Xingjuan Yu, Yifan Chu, Jing Xu, Limin Zheng

**Affiliations:** ^1^ Collaborative Innovation Center for Cancer Medicine, State Key Laboratory of Oncology in South China, Sun Yat-sen University Cancer Center, Guangzhou, P.R. China; ^2^ Department of Laboratory Medicine, The Third Affiliated Hospital, Sun Yat-sen University, Guangzhou, P.R. China; ^3^ Department of Cell Biology, Nanjing Medical University, Nanjing, P.R. China; ^4^ Department of Urology, Guangdong Provincial Key Laboratory of Malignant Tumor Epigenetics and Gene Regulation, Sun Yat-sen Memorial Hospital, Sun Yat-sen University, Guangzhou, P.R. China; ^5^ Key Laboratory of Gene Engineering of the Ministry of Education, State Key Laboratory of Biocontrol, School of Life Sciences, Sun Yat-sen University, Guangzhou, P.R. China

**Keywords:** interleukin-25, macrophages, transforming growth factor-β1, gastric cancer

## Abstract

Interleukin-25 (IL-25) is a recently identified member of the proinflammatory IL-17 cytokine family; however, its role in human tumors remains largely unknown. The aim of this study was to investigate the cellular source and clinical significance of IL-25 in gastric cancer (GC) *in situ*. The results demonstrated that macrophages (Mφs) were the primary IL-25-expressing cells (IL-25^+^) in GC *in situ*. Moreover, IL-25^+^ cells were highly enriched in the intra-tumoral (IT) region of GC tissues (*p <* 0.001). The production of IL-25 in Mφs exposed to culture supernatant from gastric cancer cell line SGC7901 *in vitro* was induced by transforming growth factor-β1, and their density in the IT region was positively associated with those of other effector immune cells, namely, CD4^+^ T cells, CD8^+^ T cells and CD103^+^T cells (*p <* 0.01). This suggested that macrophages might produce IL-25 to create an antitumor micromilieu in GC tissues. The level of IL-25^+^_*IT*_ cells was positively associated with histological grade (*p <* 0.001) and found to be an independent predictor of favorable survival (*p* = 0.024) in patients with GC after radical resection. These findings suggest that IL-25^+^_*IT*_ cells may be a novel therapeutic target in those patients.

## INTRODUCTION

Gastric cancer (GC) is the fourth most common malignancy and the second leading cause of cancer mortality worldwide [1]. The risk of developing GC can be influenced by interactions between the host and environmental factors [1]. *Helicobacter pylori* infection is the principle risk factor for the development of chronic gastric inflammation that progresses to GC [2–3]. However, the precise roles and underlying mechanisms of inflammatory components in disease progression are poorly understood.

The immune microenvironment in tumor tissues is highly organized at molecular and cellular levels. It can exhibit pro- or antitumor properties depending on the context of immune response [4–7]. Macrophages (Mφs) constitute a major component of immune cell infiltrates in nearly all tumors [8–9]. Studies have demonstrated that they could promote tumor angiogenesis, metastasis and induce T cell differentiation and activation through the production of cytokines [10–15]. Our group and others have reported that a high number of infiltrating Mφs could be correlated with both favorable and poor prognoses in different tumor types [11–19]. The interleukin-17 (IL-17) family is a subset of cytokines consisting of IL-17A-F that play crucial roles in autoimmune disease and tumor progression [20]. IL-17A is the most studied member of the IL-17 family in human tumors and has multiple cellular sources, including T cells, Mφs and mast cells [20–21]. Our earlier studies found that intra-tumoral IL-17A-producing T cells (Th17) could promote tumor progression by fostering angiogenesis in hepatocellular carcinoma [11]; whereas, mast cells expressing IL-17A in the muscularis propria predicted a favorable prognosis in esophageal squamous cell carcinoma [22]. The activated status of Mφ and the nature of IL-17-expressing cells may account for these paradoxes.

IL-25 (also known as IL-17E) is a newly identified member of the IL-17 family. It is produced in multiple cell types, including mast cells, alveolar Mφs, eosinophils and epithelial cells [23–26]. Reports have shown that IL-25 was a potent regulator of inflammation, contributing to allergic inflammation and protection against parasitic infection [23, 27–29]. IL-25 has also been implicated in tumor progression and was shown to inhibit the growth of various transplanted tumors in nude mouse models, and normal mammary epithelial-cell derived IL-25 exhibited cytotoxic activity in tumor cells [30–31].

The characterization of inflammatory components in tumor progression would contribute to our understanding of the mechanisms involved. Although previous data has suggested a potential role for IL-25 in the progression of GC [30], the nature and underlying mechanisms remain largely unknown. Therefore, the aim of this study was to examine the cellular source, distribution, clinical significance and potential role of IL-25 as a prognostic marker in GC *in situ*.

## RESULTS

### IL-25^+^ Mφs are enriched in gastric cancer tumor tissues

**Figure 1:**
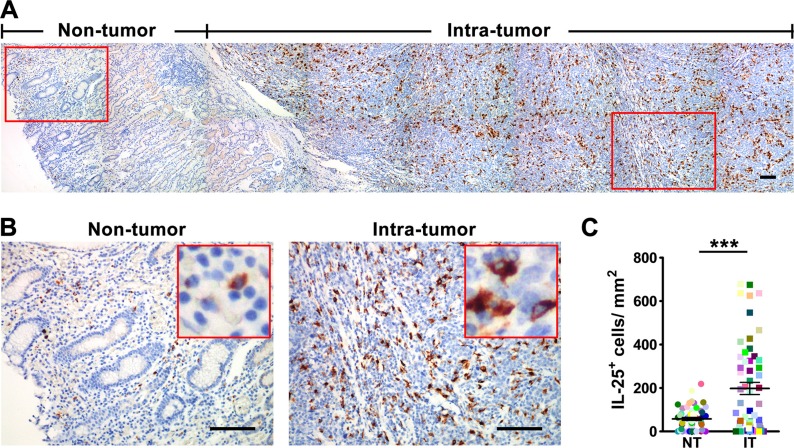
IL-25^+^ cells are enriched in the tumor tissue in gastric cancer (**A**) Immunohistochemical staining shows IL-25 in non-tumor (NT) and intra-tumor (IT) regions in gastric cancer tumor tissue. Scale bar = 100 μm. (**B**) Enlargements of the indicated areas in 1A are shown with selected areas (outlined) at a higher magnification. Scale bar = 100 μm. (**C**) Quantification confirms that the density of IL-25^+^ cells in the IT region is higher than the corresponding density in the NT region (*n* = 55). Results are expressed as means *±* SEM (bars); ***p < 0.001.

**Figure 2:**
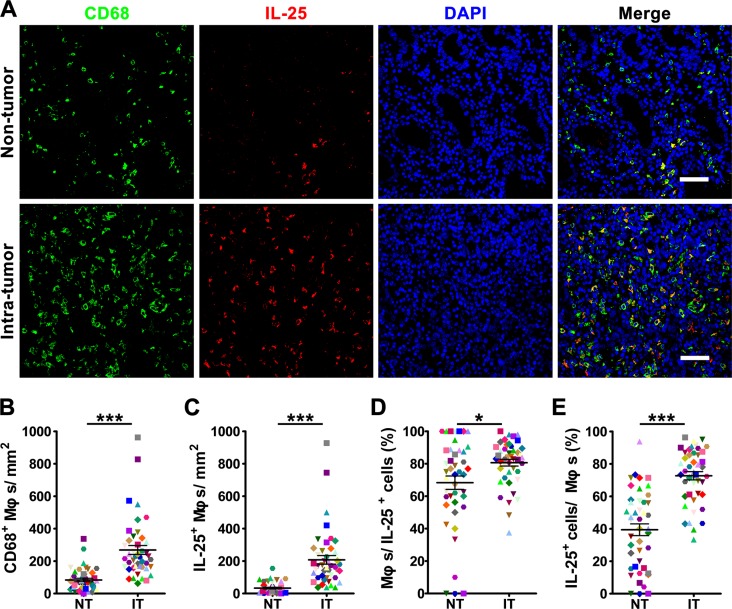
The majority of IL-25-expressing cells are produced in Mφs in human gastric cancer tissue (**A**) Double immunofluorescent staining shows the expression levels of IL-25 (red); Mφ marker CD68 (green) and co-localization of double-positive cells (yellow) in gastric cancer (GC) tissue. DAPI (blue) is used as a counterstain. Scale bar = 50 μm. (**B-E**) Comparisons between the densities of (B) CD68^+^ Mφs and (C) IL-25^+^ Mφs in non-tumoral (NT) and intra-tumoral (IT) regions of GC tissues; and the percentages of (D) IL-25^+^ cells and (E) CD68^+^ Mφs identified as double-positive (*n* = 44) compared to the total numbers of IL-25^+^ cells and CD68^+^ Mφs, respectively. Results are expressed as means *±* SEM (bars); *p < 0.05; ***p < 0.001.

To evaluate the potential role of IL-25 in tumor immunopathology, we first investigated its expression pattern in GC tissues. The presence of IL-25 protein was visualized by immunohistochemical staining in paired non-tumoral (NT) and intra-tumoral (IT) regions within 55 paraffin-embedded GC tissues. Although IL-25^+^ cells were present throughout both tissue types (Figure 1A and 1B), they were more predominant in IT tissue (IT, 197.7 *±* 28.0 cells/mm^2^; NT, 57.7 *±* 6.9 cells/mm^2^; *p* < 0.001; Figure 1C). Immunohistochemical staining levels were highest in the cytoplasm of stromal cells but were also observed in the cytoplasm of epithelial cells (Figure 1A and 1B). In addition, the IL-25^+^ stromal cells displayed irregular cell morphology and a high volume of cytoplasm (Figure 1B), suggesting they were Mφ-like cells. To test this hypothesis, double immunofluorescence was performed to identify the cellular source of IL-25 in GC tissues. Confocal microscopic analysis showed that most of the IL-25^+^ cells in both the NT and IT regions of GC tissues expressed the pan-Mφ marker CD68 (Figure 2A). Co-staining with two other Mφ markers, CD14 and CD163, demonstrated that Mφs were the principle IL-25-expressing cells in GC *in situ* (Supplementary Figure 1). Comparisons between the two regions showed that the IT region contained significantly higher amounts of CD68^+^ Mφs (IT, 268.6 *±* 27.6 cells/mm^2^; NT, 83.6 *±* 10.4 cells/mm^2^; *p* < 0.001) and IL-25^+^ CD68^+^ Mφs (IT, 207.4 *±* 26.3 cells/mm^2^; NT, 33.4 *±* 5.1 cells/mm^2^; *p* < 0.001) than the NT region (Figure 2B and 2C, respectively). Subsequent analysis showed that CD68^+^ Mφs were the principle producers of IL-25 in both IT and NT regions in GC tissues (IT, 80.6 *±* 2.1%; NT, 68.3 *±* 4.1%; *p* < 0.05; Figure 2D). In addition, the proportion of IL-25^+^ CD68^+^ Mφs relative to the total number of Mφs was significantly higher in the IT region compared to the NT region (IT, 72.7 *±* 2.5%; NT, 39.4 *±* 3.6%; *p* < 0.001; Figure 2E).

Studies in mouse models have shown that IL-25 was produced by T cells and mast cells [25, 32]. Therefore, we performed double color immunofluorescence for IL-25 using the T cell marker CD3 and the mast cell-specific enzyme tryptase (TRY). The findings showed that only a minority of IL-25^+^ cells were produced by T cells in both the IT and NT regions (IT, 0.82 *±* 0.47%; NT, 0.45 *±* 0.40%; Supplementary Figure 2A and 2C) or mast cells (IT, 3.4 *±* 1.1%; NT, 0.08 *±* 0.07%; Supplementary Figure 2B and 2D). These data demonstrated that tumor-infiltrating Mφs represented the majority of IL-25-expressing cells in GC *in situ*.

### TGF-β1 promotes production of IL-25 in macrophages *in vitro*

**Figure 3:**
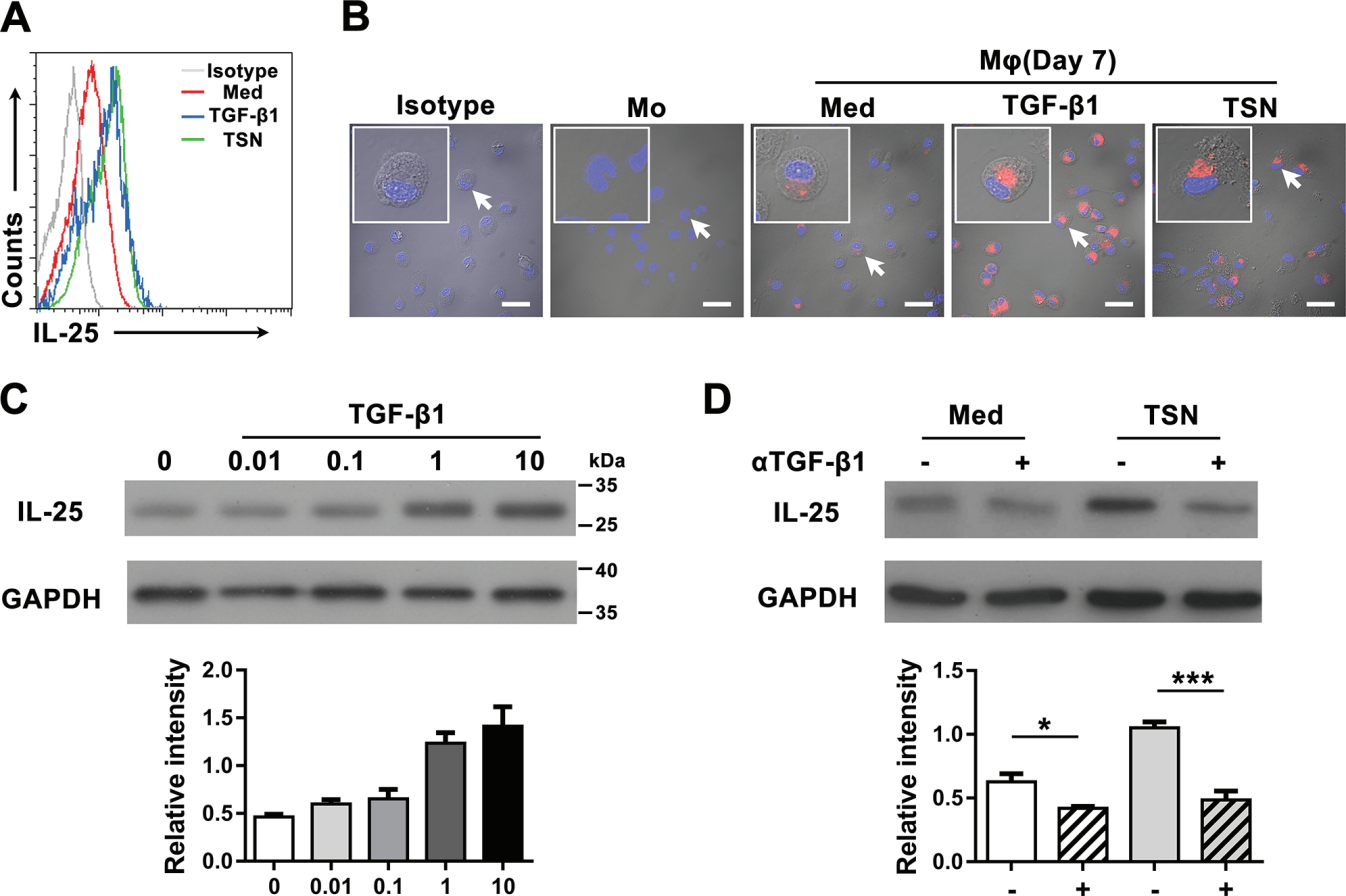
TGF-β1 promotes the production of IL-25 by Mφs in gastric cancer tissue (**A**) Flow cytometric and (**B**) immunofluorescent analyses show the expression levels of IL-25 in uncultured monocytes (Mo) and monocytes cultured in medium alone (Med), TGF-β1 (TGF-β1) or supernatant from an SGC7901 gastric cancer cell line (TSN). Scale bar = 30 μm. Negative controls were prepared using isotype-matched antibodies. (**C**) Western blotting shows the dose dependence of IL-25 expression in monocytes cultured with TGF-β1 at the indicated concentrations (ng/mL). (**D**) Western blotting shows the effect of blocking TGF-β1 in monocytes cultured with TSN: (−), untreated monocytes; (*+*), monocytes pretreated with a blocking antibody against TGF-β1. GAPDH was used as an internal control. Semi-quantitative evaluations were performed using Image J software; *p < 0.05; ***p < 0.001.

Transforming growth factor-β1 (TGF-β1) has been shown to be produced by both cancer and stromal cells in tumor tissues [33], and to positively regulate IL-25 in the human gut [34]. Therefore, we investigated the function of TGF-β1 in the production of IL-25 in GC tissue by examining freshly isolated monocytes and differentiated Mφs that had been cultured in the presence of recombinant human (rh) TGF-β1 or tumor culture supernatant (TSN) prepared from a gastric cancer cell line SGC7901. Mφs cultured in medium alone were used as controls. Flow cytometric analysis revealed that both rhTGF-β1 and TSN could effectively stimulate the production of IL-25 in Mφs (Figure 3A). However, cell immunofluorescence showed that only control Mφs, and not freshly isolated monocytes, produced IL-25. This effect was markedly upregulated by TGF-β1 and TSN (Figure 3B). Further analysis by western blotting showed that TGF-β1 stimulated IL-25 expression in Mφs in a dose-dependent manner (Figure 3C). In contrast, blocking TGF-β1 prior to culture effectively inhibited the upregulation of IL-25 protein in TSN-treated Mφs (Figure 3D). Collectively, these findings indicated that TGF-β1 induced IL-25 expression in Mφs *in vitro*.

### A high density of intra-tumoral IL-25^+^ cells is positively correlated with favorable survival in gastric cancer

To determine the potential role of tumor-infiltrating IL-25^+^ cells in the progression of GC, 106 patients who had undergone radical resection for GC were divided into high and low IL-25-expressing groups: each group was defined according to the median values of IL-25^+^ cells in the IT (IL-25^+^_*IT*_, 143 cells/mm^2^) and NT (IL-25^+^_*NT*_, 39 cells/mm^2^) regions in GC tissue. The phenotype of IL-25^+^ cells *in situ* was examined by co-staining the cells with proinflammatory and immunosuppressive markers of Mφs: CD11c and CD206, respectively [35]. The majority of IL-25^+^ cells expressed CD206, but not CD11c (Supplementary Figure 3), indicating that IL-25^+^ cells exhibited an immunosuppressive phenotype.

**Figure 4:**
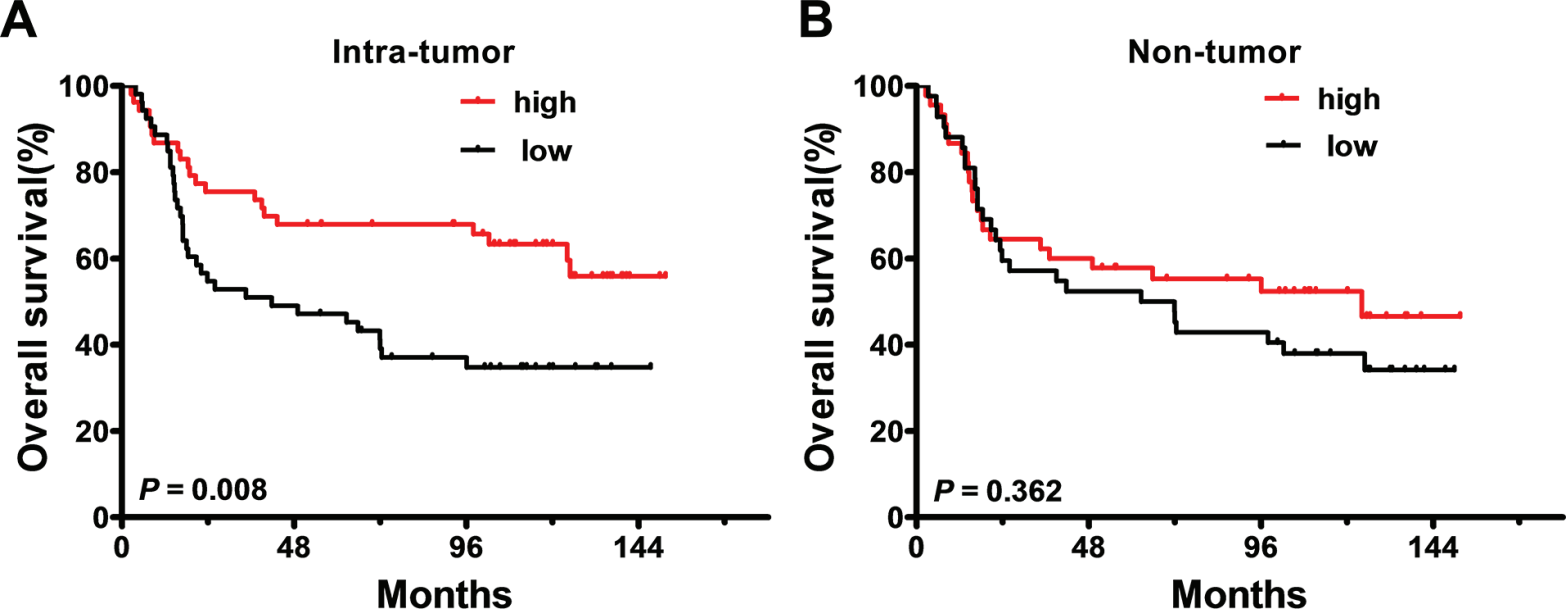
Accumulation of IL-25^+^ cells in the tumor tissue of gastric cancer is a predictor of favorable prognosis The Kaplan-Meier survival curves compare overall survival (OS) rates in gastric cancer patients (*n* = 106) with low (black) or high (red) densities of IL-25^+^ cells in the (**A**) intra-tumoral or (**B**) non-tumoral regions of the tumor tissue. Patient groups were defined according to the median values of IL-25^+^ density in each region. *p* < 0.05 was considered statistically significant (log-rank test).

Kaplan-Meier survival analysis showed a positive association between the density of IL-25^+^_*IT*_ cells and overall survival (OS) in patients with GC: patients with a high density had longer median OS than those with a low density (102.3 *vs.* 41.8 months, respectively; *p* = 0.008; Figure 4A). The 5-year OS rate was also increased in patients with a high density of IL-25^+^_*IT*_ cells (67.9%) compared to those with a low density (39.1%). In contrast, there was no correlation between the density of IL-25^+^ cells in the NT region and OS (*p* = 0.362; Figure 4B). Moreover, the density of IL-25^+^_*IT*_ cells was positively associated with histological grade (*p* < 0.001; Supplementary Table 1). We performed multivariate analysis to identify significant independent prognostic indicators of OS in patients with GC. Following the suggestion of Salama *et al.* [36], tumor depth and lymph node metastasis were not included in the multivariate model as both these variables were considered to influence TNM stage; however, all other significant clinicopathologic variables identified by univariate analysis were adopted as covariates (Table 2). The results revealed that the density of IL-25^+^_*IT*_ cells (HR = 0.5; 95% CI, 0.3–0.9; *p* = 0.024), tumor location (HR = 0.5; 95% CI, 0.3–0.9; *p* = 0.016) and TNM stage (HR = 3.7; 95% CI, 1.9–7.1; *p* < 0.001) were independent prognostic factors for OS in patients with GC. Together, these results indicated that IL-25^+^ cells inhibited tumor progression was a predictor of favorable survival in patients with GC.

**Table 1:** Clinicopathological characteristics of gastric cancer patients (*n* = 106)

Variables	No. and (%)
No. of patients	106
Age (median; range), years	59; 21–76
Gender (male / female)	76/30 (71.7/28.3)
Tumor size, cm (*≤* 4 / *>* 4)	43/62 (40.6/58.5)
Borrmann type (I / II / III / IV / other)	7/41/48/6/4 (6.6/38.7/45.3/5.7/3.8)
Tumor location (higher / middle / lower / other)	45/20/28/13 (42.5/18.9/26.4/12.3)
Tumor depth (pT1 / pT2 / pT3 / pT4)	9/15/77/5 (8.5/14.2/72.6/4.7)
Lymph node metastasis (pN0 / pN1 / pN2 / pN3)	37/47/19/3 (34.9/44.3/17.9/2.8)
TNM stage (IA / IB / II / IIIA / IIIB / IV)	4/9/36/36/15/6 (3.8/8.5/34.0/34.0/14.2/5.7)
Lauren classification (intestinal / diffuse / mixed)	70/34/2 (66.0/32.1/1.9)
Histological grade ^a^ (I / II / III / other)	1/25/55/25 (0.9/23.6/51.9/23.6)
IL-25^+^_*NT*_ cells (median; range)	39; 0–219
IL-25^+^_*IT*_ cells (median; range)	143; 0–1229

a Well-differentiated adenocarcinoma (I), moderately differentiated adenocarcinoma (II), poorly differentiated adenocarcinoma (III), other histologic type including signet ring cell and mucinous (other).

Abbreviations: pT, depth of tumor invasion; pN, lymph node metastasis; TNM, tumor-node-metastasis; NT, non-tumoral region; IT, intra-tumoral region.

**Table 2:** Univariate and multivariate analyses of factors associated with overall survival in patients with gastric cancer^a^

Variables	Univariate analysis		Multivariate analysis	
	HR^b^ (95% CI)	*p* value	HR^b^ (95% CI)	*p* value
Age, years (*>* 59 *vs. ≤* 59)	2.1 (1.2–3.7)	**0.007**	1.7 (0.9–3.2)	0.079
Gender (female *vs.* male)	0.6 (0.3–1.2)	0.140		NA
Tumor size, cm(*>* 4 *vs. ≤* 4)	2.3 (1.2–4.1)	**0.008**	1.2 (0.6–2.4)	0.596
Borrmann type (III *+* IV *+* other *vs.* I *+* II)	1.4 (0.8–2.4)	0.203		NA
Tumor location (middle *+* lower *+* other *vs.* higher)	0.5 (0.3–0.9)	**0.022**	0.5 (0.3–0.9)	0.016
Tumor depth (pT3 *+* pT4 *vs.* pT1 *+* pT2)	5.4 (1.9–15.0)	**0.001**		NA
Lymph node metastasis (pN1 *+* pN2 *+* pN3 *vs.* pN0)	2.2 (1.2–4.0)	**0.015**		NA
TNM stage (III *+* IV *vs.* I *+* II)	3.7 (2.0–6.8)	**< 0.001**	3.7 (1.9–7.1)	**< 0.001**
Lauren classification (diffuse *+* mixed *vs.* intestinal)	0.7 (0.4–1.2)	0.172		NA
Histological grade (III *+* other *vs.* I *+* II)	0.9 (0.5–1.6)	0.606		NA
IL-25^+^_*NT*_ cells (high *vs.* low)	0.8 (0.4–1.4)	0.364		NA
IL-25^+^_*IT*_ cells (high *vs.* low)	0.5 (0.3–0.8)	**0.009**	0.5 (0.3–0.9)	**0.024**

a Cox proportional hazards regression model. Variables used in multivariate analysis were adopted by univariate analysis. Significant *p* values (*<* 0.05) are shown in bold font.

b HR *>* 1, risk of death increased; HR *<* 1, risk of death decreased.

Abbreviations: pT, depth of tumor invasion; pN, lymph node metastasis; TNM, tumor-node-metastasis; NT, non-tumoral region; IT, intra-tumoral region; HR, hazard ratio; CI, confidence interval; NA, not applicable.

### IL-25^+^ cells are positively associated with effector immune cells in gastric cancer tissue

**Figure 5:**
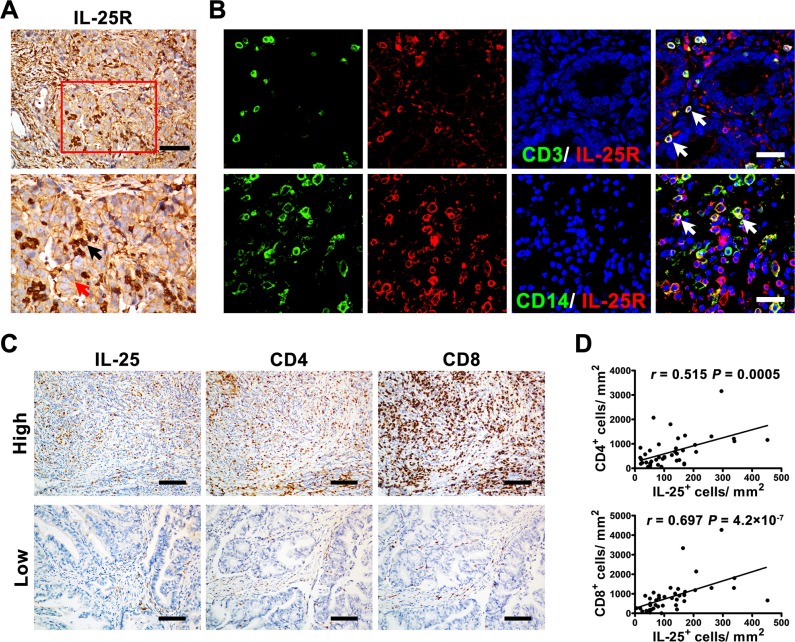
IL-25^+^ cells were positively associated with effector immune cells in gastric cancer tissue (**A**) Immunohistochemical staining shows the level and location of IL-25 receptor (IL-25R) in gastric cancer (GC) tissue. The red and black arrows indicate IL-25R^+^ tumor cells and stromal cells, respectively. Scale bar = 100 μm. (**B**) Double immunofluorescent staining with confocal microscopy shows IL-25R^+^ stromal cells in GC tissue. The white arrows indicate double-positive cells. Scale bar = 20 μm. (**C**) Representative micrographs and (**D**) quantification showing the associations between IL-25^+^ cells and other effector immune cells (CD4^+^ and CD8^+^ T cells) in GC tissue (*n* = 60). Scale bar = 100 μm. *p* < 0.05 was considered statistically significant (Spearman's rank correlation coefficient test).

We examined the expression of IL-25 receptor (IL-25R) in GC tissues to identify potential cellular targets of IL-25. Immunohistochemical staining showed that IL-25R was widely expressed in both tumor cells and stromal cells (Figure 5A and 5B). The stromal cells included CD3^+^ T cells and CD14^+^ Mφs. These findings suggested that IL-25 might either directly act on tumor cells or indirectly regulate antitumor immunity. To determine the potential regulatory function of IL-25 in immune cells, we performed Spearman's rank correlation coefficient tests to assess the correlation between the density of IL-25^+^_*IT*_ cells and tumor-infiltrating lymphocytes in GC tissues (Figure 5C and Supplementary Figure 4). The results showed that the density of IL-25^+^_*IT*_ cells was positively correlated with those of other T-cell subtypes, including CD4^+^ T cells and CD8^+^ T cells (*r* = 0.515 and *r* = 0.697, respectively; *p* < 0.01; Figure 5D) and CD103^+^ T cells and Foxp3^+^ T cells (*r* = 0.670 and *r* = 0.457, respectively; *p* < 0.01; Supplementary Figure 4). These findings suggested that the antitumor immune activity of IL-25 in GC might be mediated through the regulation of effector immune cells in the tumor tissues.

## DISCUSSION

Although there has been substantial evidence to suggest that IL-25 plays critical roles in the regulation of type-2 immune responses against parasitic infections and allergic diseases [20, 27–28], its role in tumor progression remains largely unknown. In this study, we demonstrated that Mφs, as opposed to T cells and mast cells, were the predominant producers of IL-25 in GC *in situ*. The density of IL-25^+^ cells in GC tumor tissues was associated with histological grade and could serve as a predictor of favorable prognosis in patients with GC. Investigations into the potential mechanisms showed that production of IL-25 in TSN-treated Mφs was regulated by TGF-β1 and was positively associated with effector immune cells in GC tumor tissue. These findings suggested that tumor-infiltrating Mφs might create an antitumor micromilieu by expressing IL-25 in GC tissue. This study has provided new insights into the significances of location, density and functional orientation of different immune cell populations in the progression of human carcinomas.

IL-25 can be expressed by both immune cells and non-immune cells in the context of inflammatory disorders [20]. IL-25 mRNA was first reported to be expressed by highly polarized Th2 cells and later by IgE-cross-linking mast cells [32, 37]. However, our investigations found that only a minority of all IL-25-expressing cells were located in T cells and mast cells in GC tissues, and that IL-25 predominately colocalized with Mφs-specific markers (CD68, CD14 and CD163). Statistical analysis indicated that *>* 70% of Mφs in the IT region of GC tissues expressed IL-25. These findings indicate that tumor-associated Mφs (TAM), but not T cells and mast cells, were the predominant producers of IL-25 in GC tissues *in situ*. This is consistent with previous studies that IL-25 was produced by alveolar Mφs in a rat model and subepithelial macrophage-like cells in the human gut [24, 38].

CD206 and CD11c are reported markers of immunosuppressive and proinflammatory Mφs, respectively [35]. Our results demonstrated that IL-25^+^ Mφs expressed CD206 but not CD11c in GC tissues, suggesting they exhibited an immunosuppressive phenotype *in situ*. Consistent with this observation, rhTGF-β1 significantly upregulated IL-25 in Mφs in a dose-dependent manner; in contrast, blockage of TGF-β1 abrogated this effect. It is recognized that Mφs perform different functions depending on the context of immune response within a tumor [39–40]. Despite the facts that TAM acquires a tumor-promoting immunosuppressive phenotype are reported [8, 39], our previous study has shown that a high density of Mφs concomitant with a high density of regulatory T cells (Tregs) was associated with prolonged survival in GC patients [17]. Moreover, a high level of serum TGF-β1 has shown to predict good survival in patients with GC [41]. These reports supported our observation that the density of IL-25^+^ cells in the IT region served as an independent predictor for prolonged survival in patients with GC; whereas, a similar effect was not observed in the NT region. In combination, these results suggested that IL-25^+^ Mφs with an immunosuppressive phenotype might possess antitumor properties in a GC microenvironment. This was in agreement with a study that showed M2-type chemokine CCL18 was expressed in a subset of tumor-infiltrating Mφs and that its level was associated with prolonged survival in GC patients [42].

Despite its reported antitumor effects, the molecular mechanism of IL-25^+^ cells in GC remains unclear. The biological actions of IL-25 in target cells require the expression of its receptor, IL-25R. Studies in human breast cancer have found that upregulation of IL-25R was greater in tumor cells compared to that in nonmalignant cells. In addition, IL-25 exhibited potent cytotoxic activity against breast cancer cells via IL-25R signaling pathways [31]. Although our study has indicated that GC tumor cells might express IL-25R *in situ*, and that both T cells and Mφs expressed IL-25R in GC tissue; we did not observe cytotoxic activity *in vitro* using recombinant human IL-25 in GC cell lines (data not shown). Therefore, this hypothesis will require further investigations.

It has been suggested that specific cytokines might remodel a tumor microenvironment that favors tumor eradication by regulating the recruitment and activation of other effector immune cells [22, 43–45]. Studies have also found that immune cell infiltration within the tumor microenvironment was associated with favorable outcome in patients with GC [17, 46], and that the antitumor activity of IL-25 in tumor-bearing mouse models stimulated the infiltration of eosinophils into tumor site and activation of B cells [30, 47]. In agreement with these reports, we found that IL-25^+^_*IT*_ cells were positively associated with CD4^+^ T cells, CD8^+^ T cells, CD103^+^ tissue resident memory T cells and Foxp3^+^ Tregs in the IT region of GC tissues. This suggested that IL-25 could mediate antitumor immunity by facilitating the recruitment of effector T cells to the tumor site.

In summary, we have shown that IL-25 was predominantly produced by tumor-infiltrating Mφs in the IT region of GC *in situ* and was regulated by TGF-β1 *in vitro*. Furthermore, IL-25 expression was positively associated with effector immune cells in GC and served as an independent predictor of favorable prognosis in patients with GC. Elucidating the mechanisms that selectively modulate expression of IL-25 may offer a potential strategy for the development of anticancer therapies in GC.

## MATERIALS AND METHODS

### Patients and specimens

Tissue specimens were obtained from 106 patients with pathologically confirmed gastric cancer (GC) who had undergone radical resection at the Sun Yat-sen University Cancer Center between July 1998 and December 2003. The tissue specimens were collected immediately after surgical resection and formalin fixed and paraffin-embedded (FFPE). None of the patients had distant metastasis or had undergone any neoadjuvant therapies prior to surgery. The clinicopathological characteristics of the patients are summarized in Table 1. Clinical stages were defined according to the tumor-nodes-metastasis (TNM) cancer staging system of the Union for International Cancer Control (UICC; 6th Edition, 2002). All specimens were anonymously coded in accordance with local ethical guidelines (as stipulated by the Declaration of Helsinki) and written informed consent had been obtained from the patients. The protocol was approved by the Review Board of Sun Yat-sen University Cancer Center.

Patients' follow-up data were obtained by the Cancer Center Tumor Registry, as described in previously reports [17, 22]. Of the 106 patients examined, 55 (51.9%) had died as a direct result of GC before the end of the follow-up period. Overall survival (OS) was defined as the interval between the date of surgery and the date of death or last follow-up. The range of OS was 2.6–151.5 months and the 1-, 3-, 5- and 10-year OS rates were 87.7%, 63.2%, 57.5% and 48.7%, respectively.

### Immunohistochemistry and immunofluorescence

FFPE tissue specimens taken from the advancing edge of the primary tumor were used for evaluation. Immunohistochemical staining of 5-μm-sections was carried out using a two-step protocol as previously described [17]. Briefly, sections were incubated with primary antibodies against human IL-25 (1:1000; R & D Systems; Minneapolis, MN, USA), IL-25R (1:500; LifeSpan Biosciences; Seattle, WA, USA), CD4 (1:200; Spring Biosciences; Pleasanton, CA, USA), CD8 (1:400; Thermo Fisher Scientific; Rockford, IL, USA), CD103 (1:2000; Abcam; Cambridge, United Kingdom), Foxp3 (1:200; Abcam) or control antibodies (Santa Cruz Biotechnology; Santa Cruz, CA, USA). The sections were stained using an Envision system (Dako; Carpinteria, CA, USA) as previously described [17].

Double immunofluorescent staining was carried out as previously described [22]. Briefly, FFPE sections were incubated at 4°C overnight with the following primary antibodies: mouse anti-human IL-25 (1:500; R & D Systems), IL-25R (1:200; LifeSpan Biosciences), rabbit anti-human CD3 (1:100; Thermo Fisher Scientific), CD68 (1:100; Santa Cruz Biotechnology), Tryptase (1:1000; Santa Cruz Biotechnology), CD14 (1:50; Zhongshan Biotechnology; Beijing, China) or CD163 (1:200; Epitomics; Burlingame, CA, USA). The sections were then incubated for 30 min at 37°C with a mixture of primary-antibody-matched fluorescently labeled secondary antibodies (1:500; Invitrogen; Carlsbad, CA, USA).

Cell immunofluorescence was performed by incubating fixed, permeabilized monocytes or Mφs with anti-IL-25 antibody, followed by a fluorescently labeled secondary antibody. Negative controls were generated by replacing primary antibodies with isotype-matched antibodies. Images were captured and analyzed using an FV10-ASW 1.7 Viewer (Olympus).

### Evaluation of immunohistology

To evaluate the densities of IL-25^+^, CD4^+^, CD8^+^, CD103^+^ and Foxp3^+^ cells, areas of intra-tumor (IT) and non-tumor (NT) tissue sections were screened by microscopy at low magnification (*×* 100); five representative spot images were then selected and captured at *×* 200 magnification using a computerized system that included a Digital Sight DS-Fi1 camera installed on a Nikon Eclipse 80*i* light microscope (Nikon). Cells that were stained positive were automatically counted using inForm image analysis software (Perkin-Elmer Applied Biosystems; Hopkinton, MA, USA) [48]. Cell numbers were expressed as mean *±* SEM per mm^2^.

### Quantification of immunofluorescence

Quantification was carried out as previously described [22]. The numbers of single-positive or double-positive stained cells for each treatment were manually counted in two representative images at *×* 200 magnification (0.4 mm^2^ per field) by two independent blinded assessors. The counts were used to calculate the proportions of cells in different populations; for example, the proportion of IL-25^+^ CD68^+^ Mφs compared to the total number of CD68^+^ Mφs was calculated using the formula: (number of IL-25^+^ CD68^+^ cells)/(number of IL-25^+^ CD68^+^ Mφs *+* IL-25^−^ CD68^+^ Mφs).

### Preparation of tumor culture supernatant

Human gastric cancer cell line (SGC7901) was obtained from the American Type Culture Collection (ATCC; Rockville, MD, USA). Cells were maintained in DMEM medium supplemented with 10% FBS (Gibco; Carlsbad, CA, USA). Preparation of tumor culture supernatant (TSN) was performed as previously described [13].

### Isolation and culture of human monocytes

Peripheral blood mononuclear cells (PBMC) were isolated from buffy coats acquired from healthy donors using a Ficoll density gradient as previously described [13]. The monocytes were purified from PBMCs using anti-CD14 magnetic beads (Miltenyi Biotechnology; Surrey, UK) according to the manufacturer's instructions [15]. CD14^+^ monocytes were cultured for seven days in DMEM supplemented with 10% AB serum in the presence 15% TSN or TGF-β1 (10 ng/mL; R & D Systems) or in medium alone to obtain TSN-treated Mφs, TGF-β1-treated Mφs or control Mφs (Med). TGF-β1-blocking experiments were carried out using cells pretreated for 1 h with a TGF-β1-blocking antibody (R & D Systems) or control IgG before exposure to TSN.

### Flow cytometry

Preparation of Mφs for flow cytometry was carried out as previously described [13]. The cells were stained with fluorochrome-conjugated antibodies for IL-25 (R & D Systems) or control antibodies (BD Bioscience; San Jose, CA, USA) before being analyzed by multicolor flow cytometry.

### Western blotting

The proteins were extracted following standard protocols as previously described [13]. They were separated by 10% SDS-PAGE prior to immunoblotting with antibodies against IL-25 (1:2000; R & D Systems) or GAPDH (1:5000; Santa Cruz Biotechnology) and then visualized using an ECL kit. Semi-quantitative analysis to determine the expression levels of the proteins was performed using image J software (a public domain, image processing program).

### Statistical analysis

Statistical analyses were performed using SPSS v.20.0 software (SPSS Inc.; Chicago, IL, USA). The statistical difference between groups was determined by paired *t*-test. Cumulative survival time was calculated using the Kaplan-Meier method and analyzed by the log-rank test. Multivariate analysis of prognostic factors for OS was performed using the Cox proportional hazards model. For categorical analysis, the median value was used as a cutoff to dichotomize the continuous variables. Analysis of the association between variables was conducted using Spearman's rank correlation coefficient for continuous variables and χ^2^ tests for categorical variables. Two-tailed *p* < 0.05 was considered statistically significant.

## SUPPLEMENTARY MATERIALS FIGURES AND TABLE


