# Supplementary material for: Tumor-infiltrating macrophages express interleukin-25 and predict a favorable prognosis in patients with gastric cancer after radical resection
Source: Oncotarget. 2016 Jan 31;7(10):11083–93. doi: 10.18632/oncotarget.7095 (PMC4905459; doi:10.18632/oncotarget.7095)
Supplement: Supplementary file 1 [file oncotarget-07-11083-s001.pdf]

Tumor-infiltrating macrophages express interleukin-25 and predict a favorable prognosis in patients with gastric cancer after radical resection

Supplementary Materials

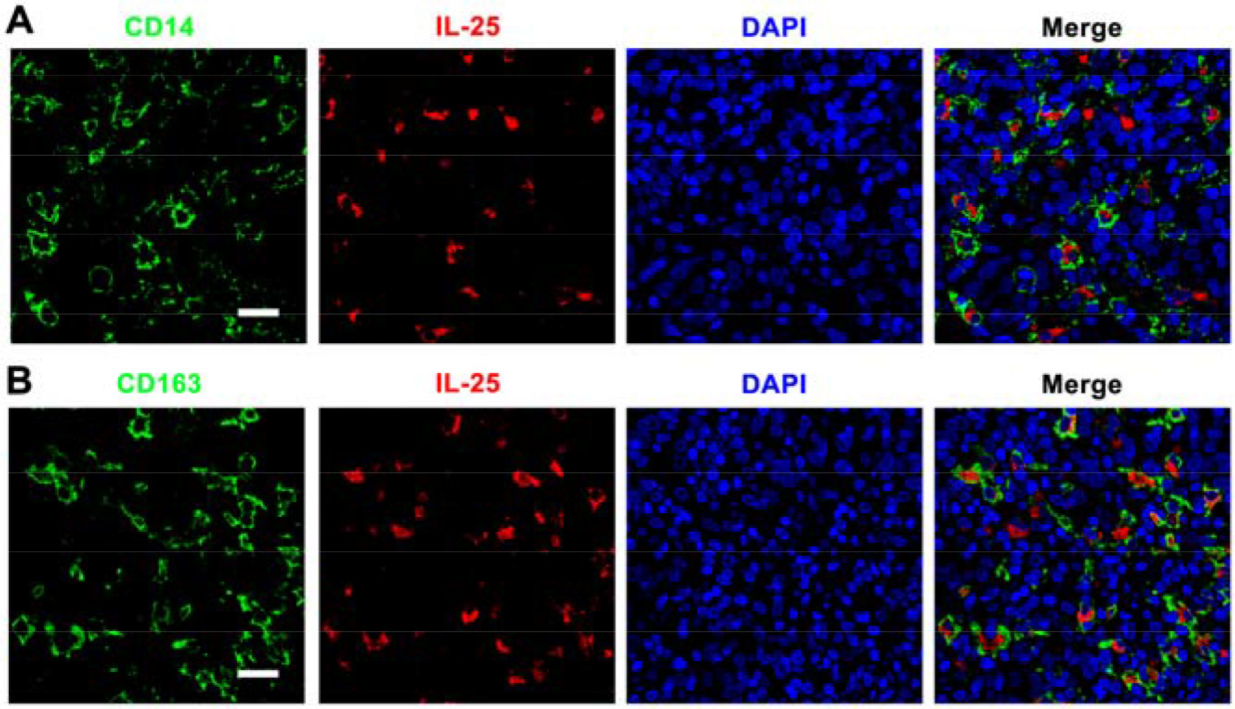

Supplementary Figure S1: Co-existence of CD14 or CD163 with IL-25 in gastric cancer tissues. Immunofluorescent staining shows IL-25 (red) with Mφ markers **(A)** CD14 and **(B)** CD163 (both green) in the intra-tumoral (IT) region of gastric cancer tissue. DAPI (blue) represents nuclei. Scale bar = 20 μm.

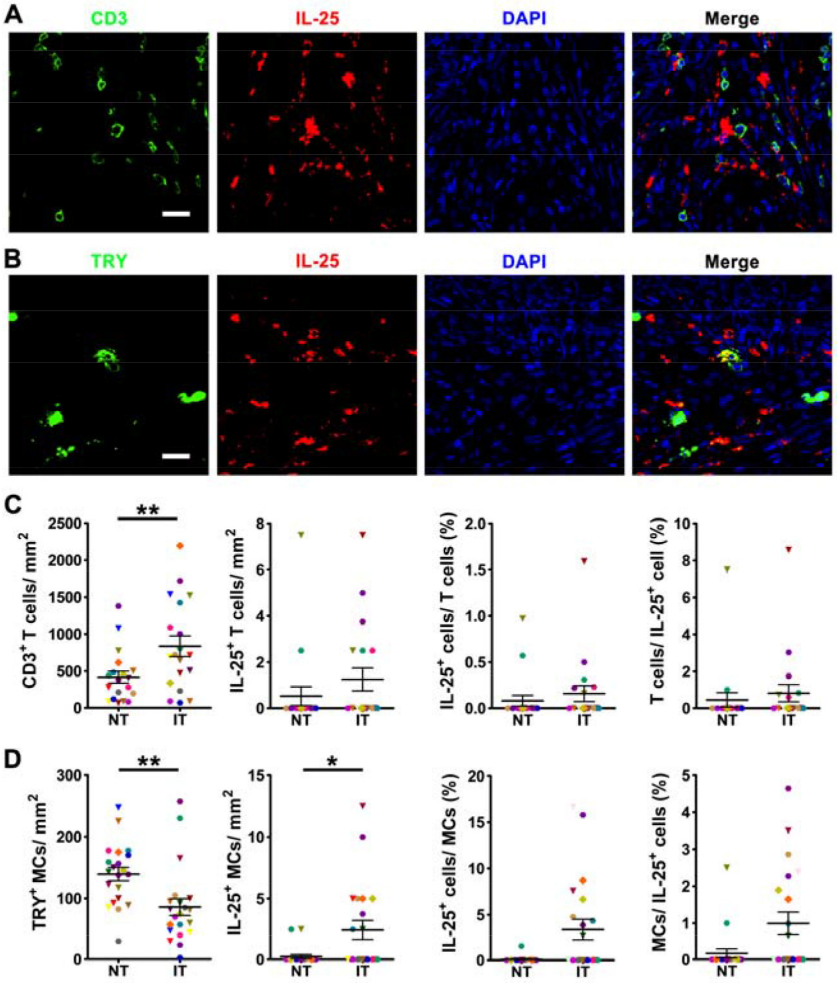

Supplementary Figure S2: Identification of the cellular sources of IL-25 in gastric cancer tissue. Double immunofluorescent staining shows IL-25 (red) with T cell marker **(A)** CD3 or **(B)** mast cell (MC) marker TRY (both green) in the intra-tumoral region of gastric cancer (GC) tissue. Scale bar = 20 μm. **(C)** Comparisons between the intra-tumoral (IT) and non-tumoral (NT) regions in GC tissue: (left to right) the densities of $CD3^+$ T cells and IL-25^+ T cells; and the percentages of double-positive cells relative to total $CD3^+$ or IL-25^+ cells (n = 19). **(D)** Left to right: the corresponding densities of TRY^+ MCs and IL-25^+ MCs; and the percentages of double-positive cells relative to total TRY^+ or IL-25^+ cells (n = 21). Results are expressed as mean ± SEM (bars). $^*p < 0.05$; $^{**}p < 0.01$.

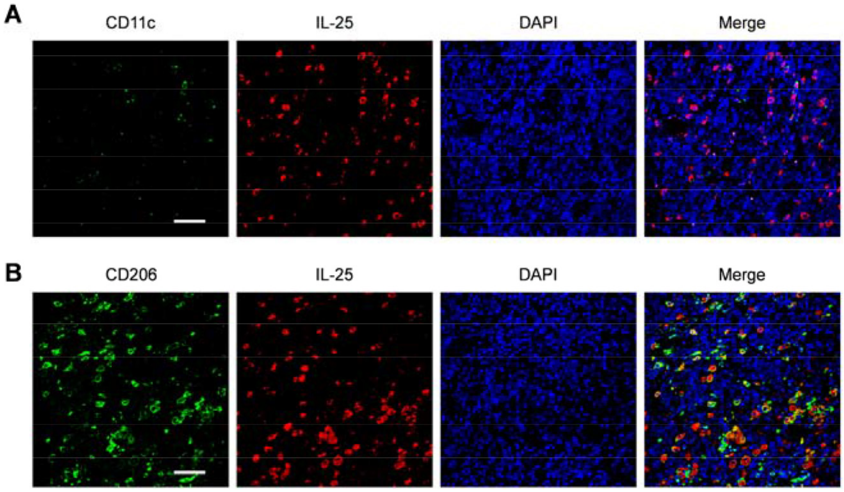

Supplementary Figure S3: Phenotypical analysis of IL-25$^+$ Mφs in gastric cancer tissue. A representative micrograph (n = 10) showing double immunofluorescence staining for IL-25 (red) and either **(A)** CD11c (green) or **(B)** CD206 (green) in the intra-tumoral region in gastric cancer tissue sections. DAPI (blue) was used as a counterstain. The results were evaluated by confocal microscopy. Scale bar = 50 μm.

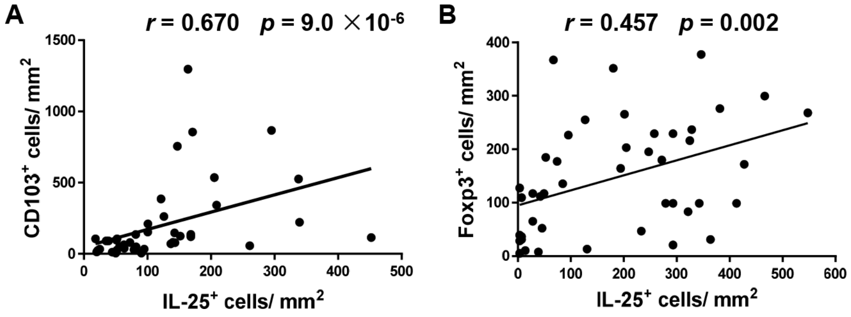

Supplementary Figure S4: The density of IL-25$^+$ cells was positively associated with T cell markers in gastric cancer tissue. Immunohistochemical quantification showing the associations between the densities of IL-25$^+$ cells and those of other immune cells in the intra-tumoral region of GC tissue: **(A)** CD103$^+$ T cells and **(B)** Foxp3$^+$ T cells. Correlations were performed by Spearman's rank correlation coefficient test; $p < 0.05$ was considered statistically significant.

Supplementary Table S1: Associations between cell densities and clinicopathological characteristics in patients with gastric cancer [a]

Variables	Intra-tumoral IL-25⁺ Mφs		
	Low [No. (%)]	High [No. (%)]	*p* value
Age, years			
≤ 59	27 (50.9)	26 (49.1)	0.846
> 59	26 (49.1)	27 (50.9)	
Gender			
Male	38 (50.0)	38 (50.0)	1.000
Female	15 (50.0)	15 (50.0)	
Borrmann type			
I + II	27 (56.3)	21 (43.7)	0.242
III + IV + other	26 (44.8)	32 (55.2)	
Tumor size, cm			
≤ 4	18 (41.9)	25 (58.1)	0.191
> 4	34 (54.8)	28 (45.2)	
Tumor location			
higher	26 (57.8)	19 (42.2)	0.169
middle + lower + other	27 (44.3)	34 (55.7)	
Tumor depth			
pT1 + pT2	8 (33.3)	16 (66.7)	0.063
pT3 + pT4	45 (54.9)	37 (45.1)	
Lymph node metastasis			
pN0	19 (51.4)	18 (48.6)	0.839
pN1 + pN2 + pN3	34 (49.3)	35 (50.7)	
TNM stage			
I + II	22 (44.9)	27 (55.1)	0.330
III + IV	31 (54.4)	26 (45.6)	
Lauren classification			
intestinal	39 (55.7)	31 (44.3)	0.101

diffuse + mixed	14 (38.9)	22 (61.1)	
Histological grade [b]			
I + II	21 (80.8)	5 (19.2)	**< 0.001**
III + other	32 (40.0)	48 (60.0)	

[a] χ^2 test. Significant *p* values (< 0.05) are shown in bold font.
Three non-significant variables are not shown: age, gender, Borrmann type.
[b] Well-differentiated adenocarcinoma (I), moderately differentiated adenocarcinoma (II), poorly differentiated adenocarcinoma (III), other histologic type including signet ring cell and mucinous(other).
Abbreviations: pT, depth of tumor invasion; pN, lymph node metastasis; TNM, tumor-node-metastasis.
